# First description of the male of *Psechrusjinggangensis* Wang & Yin, 2001 from China

**DOI:** 10.3897/zookeys.1056.68504

**Published:** 2021-08-13

**Authors:** Dan-chen Zhao, Ming-hui Fei, Xin Zeng, Yuan-hao Ying, Yong-hong Xiao, Ke-Ke Liu

**Affiliations:** 1 College of Life Science, Jinggangshan University, Ji’an 343009, Jiangxi, China Jinggangshan University Ji’an China; 2 Key Laboratory of Agricultural Environmental Pollution Prevention and Control in Red Soil Hilly Region of Jiangxi Province, Jinggangshan University, Ji’an, 343009, Jiangxi, China Jinggangshan University Ji’an China

**Keywords:** Distribution, Jiangxi Province, Jinggang Mountain National Nature Reserve, lace-sheet spiders, taxonomy

## Abstract

The male of *Psechrusjinggangensis* Wang & Yin, 2001 is described for the first time based on many specimens from its type locality, Jinggang Mountain National Nature Reserve, Ji’an City, Jiangxi province, China. Detailed illustrations, SEM images, and distribution map are given.

## Introduction

The spider family Psechridae Simon, 1890 is one of the smallest families of spiders. Currently recorded mainly from Southeast Asia, it comprises two genera, namely *Fecenia* Simon, 1887 and *Psechrus* Thorell, 1878, and 61 species (WSC 2021). They are characterised by the medium-sized to large body and a well-developed cribellum and calamistrum. While *Fecenia* construct a pseudo-orb web with a curled-leaf retreat, *Psechrus* build a conspicuously large horizontal sheet-web with a funnel-shaped retreat (with a depth from 20 cm to 1.8 m) (Jocqué and Dippenaar-Schoeman 2006; Bayer 2012). *Psechrus* usually occur in shady habitats in forests, among rocky areas, soil cracks, around cave entrances at escarpments, road cuts, or ditches. To some extent, their webs can be divided into two parts, a horizontal sheet-web and a funnel-shaped web. Horizontal sheet-webs occur in both shaded and full-sun habitats, but funnel-shaped webs only occur hidden in shaded, moist habitats. *Psechrus* feed on moths, beetles, locusts, etc. Their efficient predation, high alertness, and reproductive capacity (Xu and Wang 1983) allow them to control a particularly food-rich patch of habitat, which contributes to their wide distribution in tropical Asia, including parts of China and India (WSC 2021).

The genus *Psechrus* was erected by Thorell (1878) based on the female specimens of *Tegenariaargentata* Doleschall, 1857 from Indonesia. More details of the morphological characters of this genus were not revealed until much later, in a publication by Bayer (2012). Subsequently, the genus *Psechrus* is well understood based on the characters of 46 species, divided in eight groups: the *argentatus* group, *mulu* group, *annulatus* group, *singaporensis* group, *ancoralis* group, *himalayanus* group, *sinensis* group (which includes *Psechrusjinggangensis* Wang & Yin, 2001), and the *torvus* group (Bayer 2012). Currently, it is a diverse genus with 57 species mainly distributed in tropical Asia, and more than a quarter of these (17 species) are described from China (WSC 2021). However, three species are still only known from one sex, namely *Psechrusfuscai* Bayer, 2012, *P.jinggangensis*, and *P.kenting* Yoshida, 2009. For *P.jinggangensis*, the male has not been found since this species was described two decades ago. The male is described for the first time by us.

During the past seven years, several expeditions to the Jinggang Mountain National Nature Reserve in Jiangxi province, China, have been made by the authors, and many *Psechrus* specimens were collected. The results of these expeditions suggest that either other *Psechrus* spp., or non-*Psechrus* spp., were found. These specimens have allowed us to provide herein natural history photographs, habitus illustrations, as well as SEM and genitalia images.

## Materials and methods

We attempted to examine the holotype from Hunan Normal University, where it was reported and had been deposited (Wang and Yin 2001), but we were unable to find it, and it may be lost. Specimens were examined using a Zeiss Stereo Discovery V12 stereomicroscope with Zoom Microscope System. Additional details were studied using a Zeiss Axio Scope A1 compound microscope with a KUY NICE CCD camera. Both the male palps and female genitalia were detached from the spider body and observed in 80−85% ethanol. For SEM photographs, the specimens were kept under natural dry conditions, sprayed with gold with a small ion-sputtering apparatus ETD-2000, and photographed with a Zeiss EVO LS15 scanning electron microscope. The specimens not sprayed with gold were stored in 80% ethanol after SEM. All specimens are deposited in Animal Specimen Museum, College of Life Science, Jinggangshan University (ASM-JGSU).

All morphological measurements were taken using a stereomicroscope (AxioVision SE64 Rel. 4.8.3) and given in millimetres. The body length of each specimen does not include the spinnerets. Leg measurements are given as total length (femur, patella, tibia, metatarsus, tarsus).

Terminology of the male and female genitalia follows Bayer (2012). The abbreviations used in the text and figures are as follows:

*Body*

Cde cheliceral denticles;

OL opisthosoma length;

OW opisthosoma width;

PL prosoma length;

PW prosoma width;

TL total length.

*Eyes*

ALE anterior lateral eye;

AME anterior median eye;

AW anterior width;

MOA median ocular area;

PLE posterior lateral eye;

PME posterior median eye;

PW posterior width.

*Male palp*

Con conductor;

EB embolic base;

EBA embolic basal apophysis;

Em embolus;

Sco scopula;

SD sperm duct;

Se serrula;

St subtegulum;

Te tegulum.

*Epigyne*

CD copulatory duct;

CO copulatory opening;

EF epigynal field;

FD fertilisation duct;

LL lateral lobe;

MS median septum;

SH spermathecal head.

*Legs*

Mac macrosetae.

## Taxonomy

### Family Psechridae Simon, 1890

#### Genus *Psechrus* Thorell, 1878

##### Psechrus jinggangensis

Taxon classificationAnimaliaAraneaePsechridae

Wang & Yin, 2001

9583BD50-C56B-533A-BC95-B82A8B6E1BF3

Figures 1 , 2 , 3 , 4 , 5 , 6 , 7 , 8 , 9

Psechrus jinggangensis Wang & Yin, 2001: 334, figs 11, 12; Bayer 2012: 112, fig. 61a, b.

###### Material examined.

China – Jiangxi Province • 1 ♂ (Pse-16), Ji’an City, Jinggangshan County Level City, Ciping Town, Dajing Village, Jinggang Mountain National Nature Reserve, 26°33'21.70"N, 114°07'20.08"E, 906 m, 3 Aug. 2020, Ke-ke Liu et al. leg.; 1 pre-subadult female (Pse-17), with same data as previous; 1 pre-subadult female (Pse-1), Shiliao Cave, same locality, 3 Aug. 2019, Ke-ke Liu et al. leg.; 1 ♀ (Pse-2), with same data as previous; 1 pre-subadult female (Pse-4), with same data as previous; 1 pre-subadult female (Pse-9), with same data as previous; 1 ♂ (Pse-12), same locality, 1 Nov. 2019, Zhi-wu Chen and Dan-chen Zhao leg.; 1 pre-subadult female (Pse-14), same locality, 22 Jul.2020, Ke-ke Liu et al. leg.; 1 pre-subadult female (Pse-18), same locality, 4 Jul. 2020, Ke-ke Liu et al. leg.; 1 ♀ (Pse-22), same locality, Xiaojing Village, Longtan Scenic Spot, 26°35'02.40"N, 114°08'02.4"E, 945 m, 31 May 2014, Ke-ke Liu et al. leg.; 1 ♀ (Pse-34), with same data as previous; 1 ♀ (Pse-23), same locality, 26°35'06.0"N, 114°08'06.0"E, 989 m, 1 Jun. 2014; 1 ♀ (Pse-35), with same data as previous; 1 ♀ (Pse-38), with same data as previous; 1 ♀ (Pse-40), with same data as previous; 1 ♀ (Pse-41), with same data as previous; 1 ♀ (Pse-32), same locality, 26°35'31.20"N, 114°08'13.2"E, 934 m, 2 Aug. 2014, Ke-ke Liu et al. leg.; 1 ♀ (Pse-39), with same data as previous; 1 ♀ (Pse-36), same locality, Huangyangjie Scenic Spot, 26°38'13.2"N, 114°05'02.4"E, 898 m, 3 Aug. 2014, Ke-ke Liu et al. leg.; 1 ♂, 1 pre-subadult female (Pse-25), same locality, Jingzhu Mountain, 26°29'45.60"N, 114°04'44.4"E, 1146 m, 20 Dec. 2015, Ke-ke Liu et al. leg.; 1 pre-subadult female (Pse-46), with same data as previous; 1 ♂ (Pse-26), Luofu Town, Changguling Forest Farm, 26°50'38.4"N, 114°14'09.6"E, 602 m, 29 May 2017, Ke-ke Liu et al. leg.

###### Diagnosis.

The male of this species resembles both *Psechruschangminae* Zhang et al., 2016 (see Feng et al. 2016: 181, fig. 2b–d) and *P.clavis* Bayer, 2012 (see Bayer 2012: 121, fig. 66a–c) in having a membranous conductor with a triangular tip and the presence of five small denticles between cheliceral teeth (three in *P.changminae*). It can be separated by a more elongated tegulum along the cymbial alveolus axis (relatively expanded in *P.changminae* and *P.clavis*), an embolic base with a moderate constriction and a small apophysis (a strong constriction and a long embolic basal apophysis in *P.changminae*; an indistinct constriction and without embolic basal apophysis in *P.clavis*), and the embolus extending along the retrolateral part of the base (sub-centraxonial in *P.changminae*; sub-retrolateral in *P.clavis*). The female resembles those of *P.changminae* (see Feng et al. 2016: 181, fig. 2f, g) and *P.tingpingensis* Yin et al., 1985 (see Yin et al. 1985: 23, fig. 3C, D) in having an epigynal septum with a narrow anterior and a broad posterior part in the small globose spermathecae, but differs by the copulatory ducts separated by 1/10 of the anterior width of septum (more than 1/2 in *P.changminae*, closely touching in *P.tingpingensis*) and without a strong medial folded part (clearly present in *P.changminae* and *P.tingpingensis*), and the ratio > 1 between spermathecal head length and the spermathecal diameter (<1 in *P.changminae*; = 1 in *P.tingpingensis*) (Fig. 4C, D).

###### Description.

**Figure 1.**
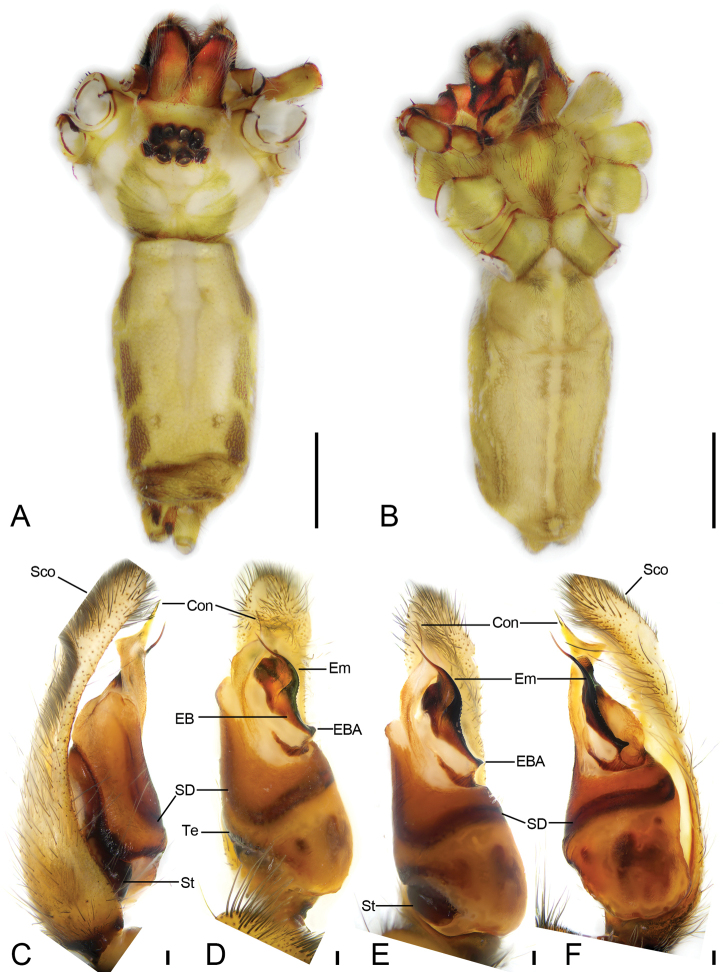
*Psechrusjinggangensis*, male (Pse-16) **A** habitus, dorsal view **B** same, ventral view **C** left palp, prolateral view **D** same, ventral view, slightly prolateral **E** same, ventral view **F** same, retrolateral view. Scale bars: 0.25 mm (**A, B**), 0.1 mm (**C–F**). Abbreviations: Con – conductor, EB – embolic base, EBA – embolic basal apophysis, Em – embolus, Sco – scopula, SD – sperm duct, St – subtegulum, Te – tegulum.

**Male. *Habitus*** as in Figure 1A, B. Total length 13.57. Prosoma (Fig. 1A) length 6.32, width 4.72, densely covered white feathery scales. Eye (Fig. 1A) sizes and interdistances: AME 0.23; ALE 0.31; PME 0.37; PLE 0.39; AME–AME 0.18; AME–ALE 0.12; PME–PME 0.29; ALE–ALE 0.91; PME–PLE 0.28; PLE–PLE 1.58; ALE–PLE 0.29; AME–PME 0.48; AME–PLE 0.71. MOA: 0.99 long; 0.71 front width, 1.04 back width. Chelicerae (Fig. 1C, D) covered by dense setae, with three promarginal teeth and four retromarginal teeth and including five small denticles between teeth. Endites (Fig. 1B), > 2× wider than their length, ectally with many long setae, median part clearly with a constriction. Labium (Fig. 1B) tongue-shaped, anteriorly with a row of strong setae, anterior margin procurved, subposterior part with a strong constriction. Sternum (Fig. 1B) oval, covered with dense setae, lateral margins with intercoxal extensions between coxae I and II, II and III, III and IV, posteriorly prolonged. Leg measurements: I 56.3 (14.73, 1.77, 16.09, 16.79, 6.92); II 43.2 (12.05, 1.95, 11.91, 12.59, 4.7); III 29.26 (8.72, 1.53, 7.28, 8.04, 3.69); IV 43.56 (11.98, 1.75, 11.56, 12.92, 5.35). Leg formula 1423. Opisthosoma length 7.46, width 3.69, hardened, with abundant short setae and feathery scales.

***Colouration and pattern*.** Prosoma, anteriorly with a brown, procurved stripe along AER, lateral margins with an arched light stripe, from PER to posteromedial part with an oval dark area, subposteriorly with four paired radial striae around fovea. Chelicerae, endites, and labium red-brown. Sternum, medially with a clear coniform brown stripe from anterior margin extending to posteromedial part. Legs from yellow to brown. Opisthosoma, dorsum from yellow to greyish black, medially with light longitudinal cardiac stripe, lateral margins with three pairs of dark-brown stripes and white stripes, with the former separated by the latter; venter with a medial, longitudinal, yellow stripe from posterior part of pedicel extending to anterior area of cribellum, and two lines of shallow depressions from bilateral part of epigastric groove extending to sub-posterior part of opisthosoma.

**Figure 2.**
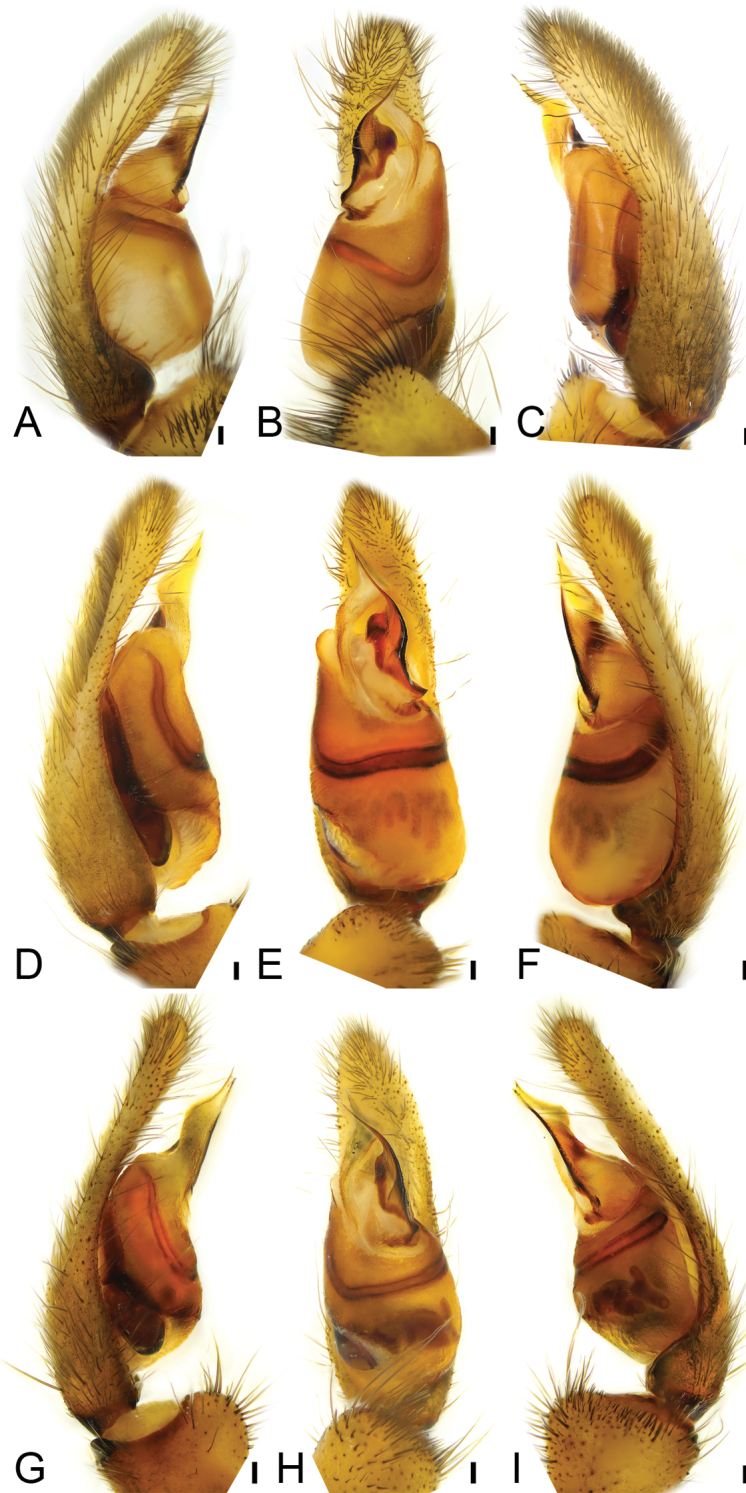
*Psechrusjinggangensis*, males. **A** right palp (Pse-12), prolateral view **B** same, ventral view **C** same, retrolateral view **D** left palp (Pse-25), prolateral view **E** same, ventral view **F** same, retrolateral view **G** left palp (Pse-26), prolateral view **H** same, ventral view **I** same, retrolateral view. Scale bars: 0.25 mm.

**Figure 3.**
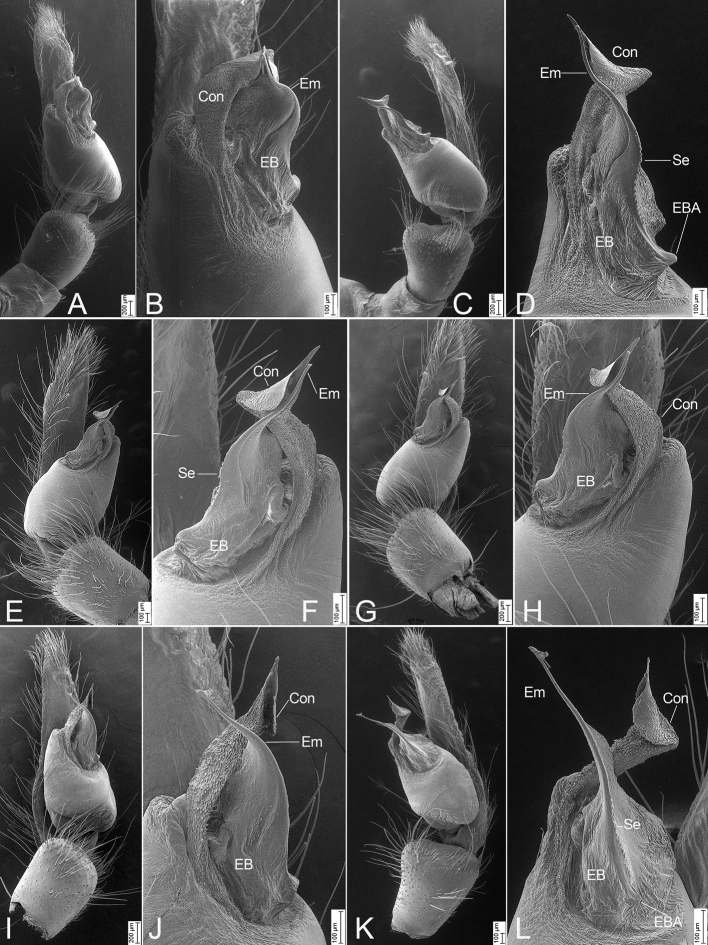
*Psechrusjinggangensis*, SEMs of males **A** left palp (Pse-16), ventral view, strongly prolateral **B** same, detail of conductor, embolic base and embolus, ventral view, strongly prolateral **C** same, retrolateral view, strongly ventral **D** same, detail of conductor, embolic base, embolic basal apophysis and embolus, retrolateral view, strongly ventral **E** right palp (Pse-12), prolateral view strongly ventral **F** same, detail of conductor, embolic base, embolic basal apophysis and embolus, prolateral view, strongly ventral **G** same, ventral view **H** same, detail of conductor, embolic base and embolus, ventral view **I** left palp (Pse-26), prolateral view, strongly ventral **J** same, detail of conductor, embolic base and embolus, prolateral view, strongly ventral **K** same, ventral view ventral view **L** same, detail of conductor, embolic base, embolic basal apophysis and embolus, retrolateral view, slightly retrolateral. Abbreviations: Con – conductor, EB – embolic base, EBA – embolic basal apophysis, Em – embolus, Se – serrula.

***Palp* (Figs 1C–F, 2A–I, 3A–D).** Palpal cymbium dorsally with very dense scopula, covering almost 1/2 of cymbium. Tegulum more than 2× longer than wide, with a clear constriction in subapical part, near the embolic base. Subtegulum strongly sclerotized, subtegular length less than tegular length in prolateral view, slightly less than posterior tegular width in ventral view. Sperm duct with V-shaped posterior part or absent in ventral view. Conductor membranous, with very dense denticles on the rough surface, slightly longer than embolus, arising from submedial part of tegulum, slightly curved retrolaterally and then pointing at the cymbial apex forming a triangular apex, with a groove on its tip. Embolic base broad, strongly sclerotized, with a small apophysis pointing retrolaterally. Embolus spine-like, extending from the subapical part of tegulum to cymbial subapex, retrolateral part strongly sclerotized, with a single row of serrula.

**Figure 4.**
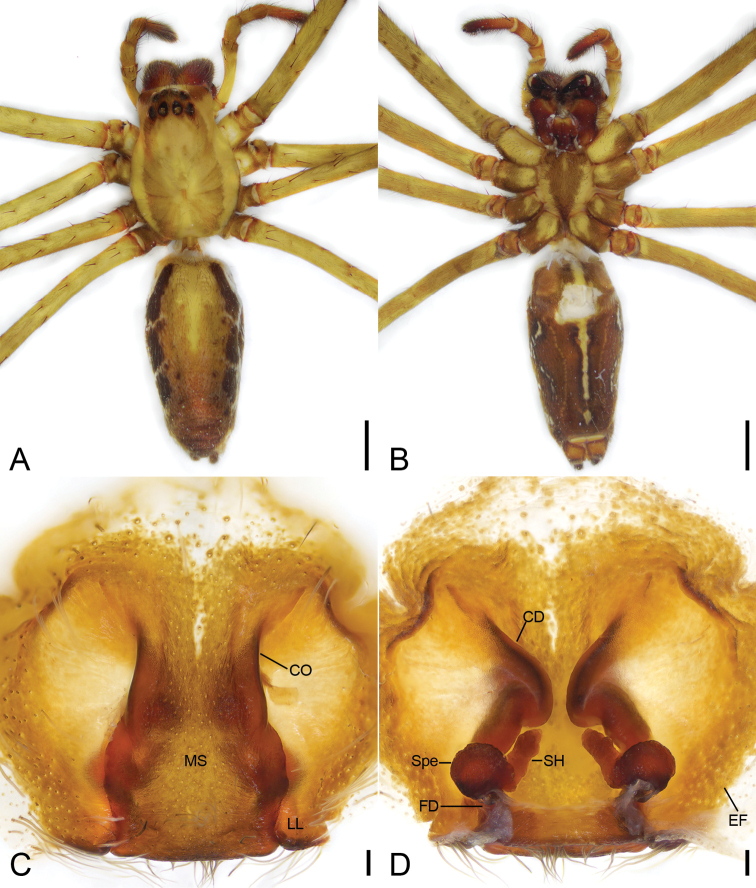
*Psechrusjinggangensis*, female (Pse-34) **A** habitus, dorsal view **B** same, ventral view **C** epigyne, ventral view **D** same, dorsal view. Scale bars: 0.25 mm (**A, B**), 0.1 mm (**C, D**). Abbreviations: CD – copulatory duct, CO – copulatory opening, EF – epigynal field, FD – fertilisation duct, MS – median septum, LL – lateral lobe, SH – spermathecal head, Spe – spermatheca.

**Female. *Habitus*** as in Figure 4A, B. As in male, except as noted. Total length 18.89. Prosoma (Fig. 4A) length 8.11, width 5.55. Eye sizes and interdistances (Fig. 4A): AME 0.4; ALE 0.43; PME 0.45; PLE 0.43; AME–AME 0.23; AME–ALE 0.11; PME–PME 0.32; ALE–ALE 1.12; PME–PLE 0.54; PLE–PLE 2.07; ALE–PLE 0.58; AME–PME 0.67; AME–PLE 0.98. MOA: 1.41 long; 0.95 front width, 1.22 back width. Chelicerae (Fig. 4A, B) with six small denticles between teeth. Leg (Fig. 4A, B) measurements: I 49.43 (13.88, 2.72, 14.42, 12.82, 5.59); II 39.82 (11.65, 2.64, 10.81, 9.75, 4.97); III 27.51 (8.75, 2.02, 6.74, 6.41, 3.59); IV 39.55 (11.63, 2.77, 9.98, 10.23, 4.94). Leg formula 1243. Opisthosoma length 10.07, width 5.16.

***Colouration and pattern*.** Darker than male. Coxae and trochanters I–IV with clear, dark, yellow–brown stripe.

**Figure 5.**
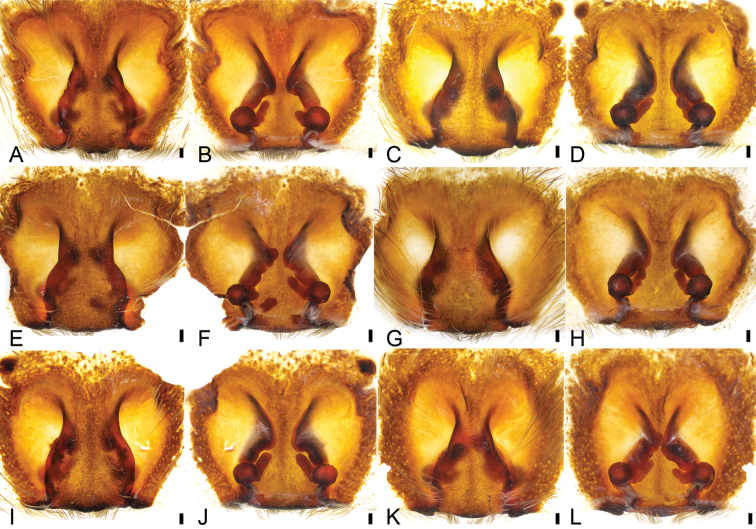
*Psechrusjinggangensis*, females **A** epigyne (Pse-35), ventral view **B** same, dorsal view **C** epigyne (Pse-36), ventral view **D** same, dorsal view **E** epigyne (Pse-37), ventral view **F** same, dorsal view **G** epigyne (Pse-23), ventral view **H** same, dorsal view **I** epigyne (Pse-32), ventral view **J** same, dorsal view **K** epigyne (Pse-33), ventral view **L** same, dorsal view. Scale bars: 0.1 mm.

**Figure 6.**
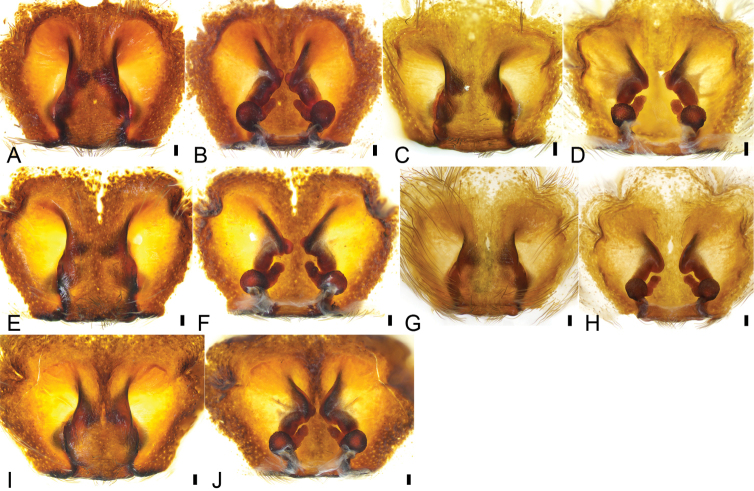
*Psechrusjinggangensis*, females **A** epigyne (Pse-40), ventral view **B** same, dorsal view **C** epigyne (Pse-38), ventral view **D** same, dorsal view **E** epigyne (Pse-39), ventral view **F** same, dorsal view **G** epigyne (Pse-22), ventral view **H** same, dorsal view **I** epigyne (Pse-41), ventral view **J** same, dorsal view. Scale bars: 0.1 mm.

**Figure 7.**
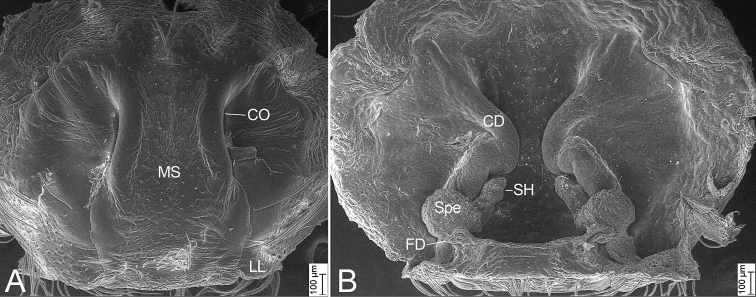
*Psechrusjinggangensis*, SEMs of females **A** epigyne (Pse-34), ventral view **B** same, dorsal view. Abbreviations: CD – copulatory duct, CO – copulatory opening, FD – fertilisation duct, MS – median septum, LL – lateral lobe, SH – spermathecal head, Spe – spermatheca.

***Epigynum* (Figs 4C, D, 7A, B).** Median septum, lateral margins strongly sclerotized, anterior part covered the copulatory openings, anterior width slightly less than 1/2 of maximum epigynal width, subposterior width almost as long as 1/2 of maximum epigynal width, posterior part with a clear constriction. Copulatory openings large, converging to median. Lateral lobe with a slightly sclerotized posterior margin. Copulatory ducts, anterior part bugle-shaped, medial part S-shaped, spiralling backwards and extending posterolaterally, posterior part tube-shaped, connecting with the spermathecae. Spermathecae globose, medially connecting with spermathecal heads, separated by < 2× spermathecal diameter. Spermathecal heads with many pores on surface, relatively long, extending forward from mesial part of spermathecae to the turn of copulatory duct. Fertilisation duct relatively broad, medially located at the spermathecae.

###### Habitat and biology.

Specimens occurred near both sides of a ditch and at the entrance areas of caves. They were usually collected by hand or by sweeping in microhabitats which included as stones, soil cracks, and plant roots. These spiders usually hang upside down on lace-sheet webs or rests on tube-shaped entrances of its web. At a slightest disturbance, they run back to their retreat with extreme speed, or they fall to the ground to feign death. This species is not easy to catch by hand and with tools.

**Figure 8.**
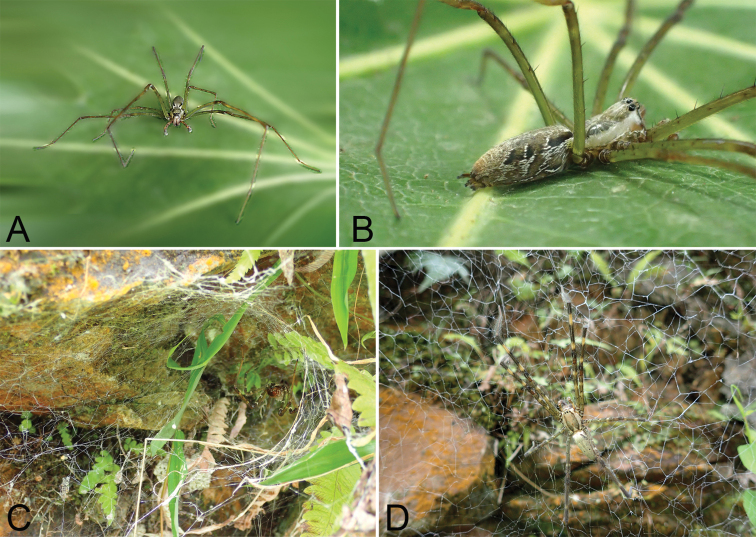
Photographs of living specimens of *Psechrusjinggangensis* from Jinggang Mountain National Nature Reserve. **A, B** male **C, D** female.

###### Distribution.

**Figure 9.**
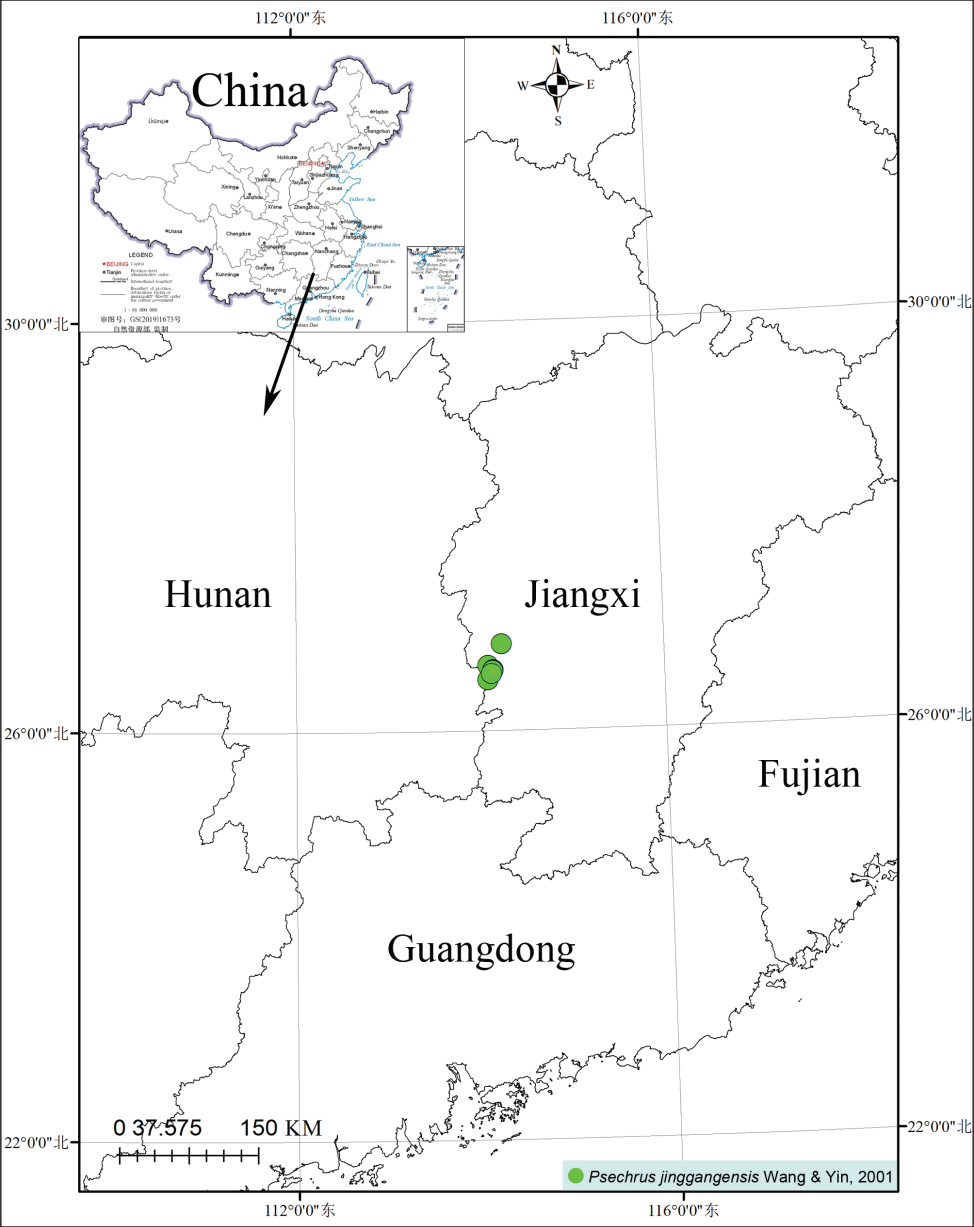
Distribution of *Psechrusjinggangensis* in China.

Known only from Jiangxi Province, China (Fig. 9). This species is widely distributed in Jinggang Mountain National Nature Reserve in Jiangxi Province, where the nature reserve abuts Yanling County in Hunan Province.

###### Variability.

Four males and 20 females were collected by us from Jinggang Mountain National Nature Reserve. Our detailed study of these specimens reveals that they differ in the number of denticles present between the cheliceral teeth, as well as in their body sizes, eye sizes, eye interdistances, and leg length (Table 1). Variability was also observed in the male palp (Figs 1C–F, 2, 3) and the epigynal field (Figs 4C, D, 5–7), such as dense scopula covering from 1/2 to 2/3 of cymbium (almost 1/2 (Pse-12 and Pse-16), > 1/2 (Pse-25), > 2/3 (Pse-26)), embolic basal apophysis from indistinct (Pse-12) to distinct (Pse-16, Pse-25, and Pse-26), anterior part of embolic base from unexpanded (Pse-26) to expanded (Pse-16, Pse-12 and Pse-25), anterior part of female epigynal septum from narrow (Pse-33) to relatively broad (Pse-34, Pse-35, Pse-36, Pse-37, Pse-23, and Pse-32), each copulatory duct from touching (Pse-33) to clearly separated (others) and with (Pse-34, Pse-35, Pse-37, Pse-23, Pse-32 and Pse-33) or without a strong turn (Pse-36), and spermathecal heads extending to the turn of copulatory ducts (Pse-34, Pse-23, and Pse-32) or not (Pse-35, Pse-37, and Pse-33). The variability observed in above may be the result of environmental factors, such as temperature, food, elevation, or habitat. Those specimens collected from stones usually had larger body sizes than specimens from other microhabitats.

**Table 1.** The variability in the number of small denticles between cheliceral teeth, body sizes (in mm), eye sizes, eye interdistances (in mm), and leg length (in mm) of *Psechrusjinggangensis*, males and females.

	Male (*n* = 4)	Female (*n* = 7)
CDe	4–7	6–9 (*n* = 8)
TL	9.67–13.57	13.16–20.82
PL	4.77–6.32	5.68–9.44
PW	3.72–4.72	4.57–6.41
OL	5.21–7.48	7.96–12.27
OW	2.23–3.74	4.05–7.45
AME	0.23–0.33	0.34–1.32
ALE	0.31–0.4	0.42–0.55
PME	0.35–0.41	0.43–0.55
PLE	0.35–0.45	0.42–0.59
AME-AME	0.11–0.18	0.18–0.24
AME-ALE	0.04–0.12	0.06–0.16
PME-PME	0.17–0.29	0.3–0.38
PME-PLE	0.26–0.28	0.38–0.54
AME-PME	0.39–0.48	0.56–0.69
AME-PLE	0.57–0.71	0.77–1.07
ALE-ALE	0.78–0.91	1.02–1.32
PLE-PLE	1.46–1.58	1.75–2.36
ALE-PLE	0.27–0.36	0.42–0.58
MOA L	0.94–1.17	1.18–1.59
MOA AW	0.67–0.75	0.79–1.06
MOA PW	0.88–1.04	1.15–1.41
Leg I	45.82–56.3 (*n* = 3)	37.66–49.76
Leg II	35.7–43.2	27.4–40.86
Leg III	22.75–29.26	21.84–40.75
Leg IV	35.71–43.56	28.78–39.55

## Discussion

Although we have not provided evidence such as microscopic examination of the female holotype or DNA analyses to certify the specimens are conspecific with the female holotype of *Psechrusjinggangensis*, the male specimens were inferred as conspecific with the female holotype of this species based on a variety of reasons discussed below. Firstly, many specimens were collected several times at the type locality in the past seven years and their genitalia are consistent with the descriptions by Wang and Yin (2001) and Bayer (2012), although with variability within the species. Secondly, many living female specimens were close to the males when observed at their natural habitat of Jinggang Mountain National Nature Reserve; however, their copulation was observed only once, on a female lace-sheet web. In addition, the females and males are widely distributed in this area and relatively easily collected by using hand and sweeping methods during their mating period.

## Supplementary Material

XML Treatment for Psechrus jinggangensis
